# Autistic Traits and Empathy in Children With Attention Deficit Hyperactivity Disorder, Autism Spectrum Disorder and Co-occurring Attention Deficit Hyperactivity Disorder/Autism Spectrum Disorder

**DOI:** 10.3389/fnins.2021.734177

**Published:** 2021-11-23

**Authors:** Stefania Aiello, David Vagni, Antonio Cerasa, Elisa Leonardi, Cristina Carrozza, Francesca Famà, Agrippina Campisi, Flavia Marino, Rosamaria Siracusano, Maria Ausilia Alquino, Francesco Mainiero, Eva Germano, Gennaro Tartarisco, Giovanni Pioggia, Antonella Gagliano, Liliana Ruta

**Affiliations:** ^1^Institute for Biomedical Research and Innovation (IRIB), National Research Council of Italy (CNR), Messina, Italy; ^2^S. Anna Institute, Crotone, Italy; ^3^Pharmacotechnology Documentation and Transfer Unit, Preclinical and Translational Pharmacology, Department of Pharmacy, Health Science and Nutrition, University of Calabria, Arcavacata, Italy; ^4^Division of Child Neurology and Psychiatry, Federico II University Hospital Naples, Naples, Italy; ^5^Division of Child Neurology and Psychiatry, Department of the Adult and Developmental Age Human Pathology, University of Messina, Messina, Italy; ^6^Department of Psychology, Faculty of Medicine and Psychology, Sapienza University of Rome, Rome, Italy; ^7^Child and Adolescent Neuropsychiatry Unit, Department of Biomedical Sciences, University of Cagliari, Cagliari, Italy

**Keywords:** autistic traits, empathy, ADHD, ASD, gender

## Abstract

Attention Deficit Hyperactivity Disorder (ADHD) and Autism Spectrum Disorders (ASD) are two of the most represented neurodevelopmental conditions in childhood. The diagnostic shift introduced by the DSM-5, allowing a combined diagnosis of ADHD and ASD, poses different clinical challenges related to diagnostic overshadowing, accuracy of clinical judgment and potential delay in an ASD diagnosis in children presenting with ADHD. Here we tried to disentangle the clinical phenotype and specificity of the two co-occurring conditions in relation to autism traits and empathy, by comparing children with ASD with and without comorbid ADHD with children presenting ADHD only and children with typical development. The child versions of the Autism Quotient (C-AQ) and Empathy Quotient (C-EQ) were administered to a total sample of 198 male children between 6 and 14 years old with age appropriate language skills and normal intelligence. Univariate analysis demonstrated no significant differences in the C-AQ total and subscale scores as well as the C-EQ between children with ASD and children with ASD + ADHD, while children with ADHD alone presented an intermediate phenotype between ASD and TD. Furthermore, a receiver operating characteristic (ROC) analysis was applied to discriminate among the different phenotypes. We found that the C-AQ and C-EQ were accurate at distinguishing with satisfactory reliability between: (a) ASD vs. non- ASD (N-ASD) groups comprising both ADHD and TD children (Area Under the Curve AUC 88% for C-AQ and 81% for C-EQ); (b) ASD and TD (AUC 92% for C-AQ and 95% for C-EQ); (c) ASD and ADHD (AUC 80% for C-AQ and 68% for C-EQ). Our data confirm the reliability of the C-AQ and C-EQ as behavioral markers to differentiate ASD (regardless of comorbid ADHD) from an ADHD condition and TD. Interestingly, in our sample an ADHD condition does not increase the severity of the clinical phenotype in terms of autism traits distribution and empathy, suggesting that the psychological measures detected by the two quantitative instruments are independent of ADHD traits. This evidence will contribute to the translational efforts in developing better tailored treatments and preventive strategies.

## Introduction

The Diagnostic and Statistical Manual of Mental Disorders fifth edition (DSM-5) (American Psychiatric Association [APA], 2013) introduced a new conceptualization of Neurodevelopmental Disorders, providing a fundamental shift from a categorical to a dimensional system of diagnosis (Hudziak et al., 2007; Jones, 2012). In the DSM-5, remarkable modifications affected one of the currently most represented neurodevelopmental conditions in childhood: Autism Spectrum Disorder (ASD).

The major changes in the ASD diagnostic category were: (i) the rearrangement of the three core domains down to two core domains (social communication and restricted and repetitive behaviors); (ii) the removal of sub-diagnoses (Autistic Disorder, Asperger Syndrome, Pervasive Developmental Disorder Not Otherwise Specified, Disintegrative Disorder) in favor of a dimensional approach of symptoms’ severity; and (iii) the possibility of a comorbid diagnosis of ADHD.

Within the autism phenotype, restricted repetitive behaviors have been linked to cognitive rigidity (South et al., 2007; Lemonda et al., 2012; Faja and Nelson Darling, 2019), hyperfocus and difficulties in predictive coding (van de Cruys et al., 2014). Different studies have replicated an impairment in executive functions (EF) such as verbal working memory, inhibition and quick visual scanning in the ASD population (Rommelse et al., 2015; Operto et al., 2021). Furthermore, EF is known to self-regulate emotional and empathic abilities, also referred to as “Hot Executive functions” (Yang et al., 2011), which result impaired in children with autism (Harmsen, 2019), with significantly higher deficits in emotional regulation (ER) than other neurodevelopmental and psychiatric disorders (Samson et al., 2012; Joshi et al., 2018). Additionally, difficulties in controlling emotions often result in internalizing problems such as anxiety and depression (Gadow et al., 2012; Operto et al., 2021).

Impairments in EF and self-regulation represent core neuropsychological features of ADHD. Interestingly, poor quality of social perspective’s assumptions, impaired social cognition, lack of pragmatic language and empathy have also been observed in children with ADHD (Marton et al., 2009; Bora and Pantelis, 2016; Lasmono et al., 2021). With respect to empathy subdomains, it has been reported that ADHD is mainly characterized by a deficit in affective empathy (sharing and responding to another individual’s emotions) rather than to its cognitive domain (understanding another person’s perspective) (Abdel-Hamid et al., 2019; Cristofani et al., 2020; Fantozzi et al., 2021) as it is usually observed in ASD (Rueda et al., 2015). The emotional and behavioral profile in ADHD children results in both internalizing and externalizing problems such as mood and anxiety disorders, aggressive and/or oppositional behaviors, with consequent difficult peer relationships (Nijmeijer et al., 2008; Harkins et al., 2021). Furthermore, many children with ADHD also show hyperfocus on topics that meet their personal interests (Groen et al., 2020) and demonstrate difficulties in processing speed and working memory (Molavi et al., 2020; Operto et al., 2021).

Previous evidence confirmed the symptoms overlap between ADHD and ASD, with 18–50% of children with ADHD presenting with clinical levels of ASD symptoms (Ronald et al., 2008; Kochhar et al., 2011; Van Der Meer et al., 2012; Grzadzinski et al., 2016) and conversely, 40–70% of children with ASD displaying co-occurring ADHD (Salazar et al., 2015; Joshi et al., 2017; Antshel and Russo, 2019).

Diagnostic accuracy may be even more challenging when ASD and ADHD co-occur. In fact, the neuropsychological and behavioral overlapping between the two conditions poses important questions related to diagnostic overshadowing and need to be in depth investigated to explain possible shared etiologies (Antshel and Russo, 2019). Furthermore, studying the co-occurrence of the two conditions at early stages is crucial to be aware of how they evolve and, consequently, how their pathways vary along with child development. This longitudinal perspective is fundamental to enhancing targeted treatment. Previous findings suggest that, in the socio-communicative domain, ADHD lies intermediately between ASD and control groups (Grzadzinski et al., 2011; van de Cruys et al., 2014; Salley et al., 2015; Groen et al., 2018). ADHD and ASD could then belong to the same continuum and represent two different manifestations of a common spectrum disorder. A recent study showed that compared to only ASD, an ADHD comorbidity was associated with reduced cognitive task performance (Mansour et al., 2021). Other studies demonstrated that an ASD + ADHD condition increased deficits in social interaction and was associated with lower adaptive functioning, higher anxiety, lower empathy (Colombi and Ghaziuddin, 2017; Shephard et al., 2019; Dellapiazza et al., 2021) and a poorer quality of life (Gadow et al., 2009; Sikora et al., 2012; Joshi et al., 2017). Furthermore, with regard to their emotional and behavioral profile, a recent study has demonstrated that children with comorbid ASD and ADHD result with higher externalizing problems than ASD alone, and lower externalizing symptoms than children with ADHD alone (Carta et al., 2020). Similarly, children with a diagnosis of ADHD who displayed higher autistic traits showed lower cognitive and social skills and the presence of autistic traits determined a more severe outcome (Kotte et al., 2013; Green et al., 2016; Joshi et al., 2020; Sesso et al., 2020).

Based on existing literature we aimed to disentangle the clinical phenotype and specificity of the two co-occurring conditions in relation to autistic traits and empathy, by comparing children with mild to moderate ASD, with normal intelligence and functional language, with and without comorbid ADHD, with children presenting with ADHD only and typically developing (TD) children. Specifically, we intend to explore if comorbid ADHD is associated with an increase in the expression of autistic traits and a decrease of empathy as detected by two dimensional measures such as the child versions of the Autism-Spectrum Quotient (C-AQ) (Auyeung et al., 2008; Ruta et al., 2012) and the Empathy Quotient (C-EQ) (Auyeung et al., 2009). In particular, we are interested in investigating whether the C-AQ and C-EQ are able to distinguish between ASD with or without ADHD and ADHD alone, as well as against typical development.

## Materials and Methods

### Subjects

All the children presenting a clinical diagnosis of ADHD and/or ASD were assessed at the clinical facilities of the National Research Council of Italy (IRIB—CNR) and the Polyclinic University Hospital “AOU G. Martino” both located in Messina, Italy, while TD children were recruited in three big mainstream schools in the metropolitan area of Messina. Clinical diagnosis was examined by medical records and was then confirmed by an experienced child neuropsychiatrist (A.G., E.G., and R.S.) and a chartered clinical psychologist of the team (S.A., E.L.) according to the DSM-5 criteria, with the support of the Autism Diagnostic Observation Schedule-2nd edition (ADOS-2, Module 3) and the parent version of the Conners scale-3rd edition, respectively. All the ASD children included in the study, received a diagnosis of ASD without disorder of intellectual development and with no impairment of functional language (6A02.0) according to ICD-11. All the children had an IQ between 70 and 130 (mean = 98.7, *SD* = 14), assessed using the Wechsler Intelligence Scale for Children-4th edition (WISC-IV).

Exclusion criteria included children with (1) intellectual disability; (2) syndromic (secondary) autism; (3) no fluent language; (4) level 3 (severe) at the DSM-5 severity classification, because for those children many questions on the C-AQ and C-EQ were not applicable or a positive answer would capture difficulties collateral to ASD and not nuclear to it (e.g., “when my child talks on the phone, he doesn’t know when it’s his turn to talk”).

The study was reviewed and approved by the Ethics Committees of CNR (ethical clearance, 01.08.2018) and parents of the children included in the study provided their written informed consent.

### Behavioral Measures

The child version of the Autism Spectrum Quotient (C-AQ) and Empathy Quotient (C-EQ) were used to assess Autistic Traits (ATs) and empathy in all the children. The C-AQ and C-EQ were originally developed to study dimensional autistic traits and empathy as a continuum. The C-AQ (Auyeung et al., 2008; Ruta et al., 2012) is a 50-items parent-report questionnaire to assess five areas associated with autism: social skills, communication, imagination, attention switching, and attention to details. It is an adapted version of the AQ for adults, a reliable tool able to identify autistic traits in the general population also at a subthreshold level, discriminating high functioning autism from non-autistic individuals. Items specifically map into the clinical features of autism (ex. She/he prefers to do things the same way repeatedly). Answers are measured with a Likert scale, from 1 to 4 (Definitely Disagree, Slightly Disagree, Slightly Agree, Definitely Agree) with higher scores indicating more autistic traits and behaviors.

The C-EQ (Auyeung et al., 2009) is a 27-items parent-report questionnaire to measure the degree of empathy expressed in real life situations, experiences, and interests. It is an adapted version of the EQ for adults, a trustworthy instrument to evaluate individual behaviors into situations where empathizing skills are required (ex. My child often doesn’t understand why some things upset other people so much). Items are presented with a Likert format from 1 to 4 (Definitely Disagree, Slightly Disagree, Slightly Agree, Definitely Agree) with higher scores indicating higher empathic skills. The EQ demonstrated a significant sex difference in the general population (with boys usually resulting as less empathizing than girls) and a negative correlation with autistic traits.

### Statistical Analyses

All statistical analyses were conducted using the SPSS Statistics Release 26.0 (IBM SPSS, New York, NY). A two-way ANOVA was performed separately for the C-AQ and the C-EQ. We performed a univariate analysis of variance (ANOVA) to verify between-group differences in the total scores of the C-AQ and C-EQ; the four diagnostic groups were used as dependent variables, the C-AQ and C-EQ were used as independent variables. Levene’s Test was used in ANOVA and Box’s Test in MANOVA to test the equality of error variances. Given that the groups size was uneven, in case of unequal variances a robust Bias-corrected accelerated (BCa) Bootstrap with 1,000 samples, stratified by group, will be used (Krishnamoorthy et al., 2007). Age and IQ were not matched across all the groups, hence all the analyses were controlled for these measures.

Furthermore, we executed a MANOVA, with the same factors and covariates, to analyze between-group differences in AQ subscales. Multiple comparisons were performed by applying Sidak correction. Pillai’s Trace was used to test the null hypothesis.

A receiver operating characteristic (ROC) curve of the C-AQ and C-EQ total scores was calculated to plot sensitivity and 1-specificity in the whole ASD group (ASD+ and ASD−) vs. N-ASD group (ADHD and TD together). The area under the curve (AUC) is a measure of the overall predictive validity, where an AUC = 0.50 indicates a random prediction of the independent variable, AUC > 0.70 indicates fair validity and AUC > 0.90 indicates excellent validity. Potential cut-off scores on the AQ and EQ for differentiating between children with and without ASD were also evaluated using ROC analysis to determine the cut-point corresponding to the best combination of sensitivity and specificity.

## Results

### Group Differences on the Child Versions of the Autism Quotient and Empathy Quotient Total Scores

A total sample of 198 male children aged between 6 and 14 years old (mean = 8.8, *SD* = 2.2) has been recruited and tested in the study. *N* = 77 children presented a diagnosis of ASD only (ASD−); *n* = 24 children received a diagnosis of ASD and ADHD (ASD+); *n* = 33 children had ADHD only and *n* = 64 children had typical development (TD) (see Table 1).

**TABLE 1:** Demographic data and explored variable comparison for the sample.

Variable	ASD− (*N* = 77)	ASD+ (*N* = 24)	ADHD (*N* = 33)	TD (*N* = 64)	Statistics	*Post hoc*
Age (95% C.I.)	9.11 (8.64, 9.60)	8.46 (7.98, 8.89)	8.51 (7.95, 9.09)	9.83 (9.41, 10.23)	*F*_(3, 197)_ = 5.631, *p* = 0.001	ASD− = TD; TD > ASD+ = ADHD
IQ (95% C.I.)	97.6 (94.5, 100.8)	106.6 (100.7, 111.9)	101.0 (96.4, 105.7)	96.6 (93.3, 99.8)	*F*_(3, 197)_ = 3.676, *p* = 0.013	ASD + > TD; ASD− = ADHD = TD
AQ tot (95% C.I.)	88.2 (83.1, 93.1)	94.0 (87.7, 100.0)	68.7 (64.3, 72.6)	54.4 (50.6, 57.9)	*F*_(3, 192)_ = 52.0, *p* < 0.001, η_*p*_^2^ = 0.448	ASD + = ASD− > > ADHD > > TD
AQ soc (95% C.I.)	17.7 (16.6, 18.8)	18.6 (16.6, 20.6)	11.2 (9.5, 12.9)	7.79 (6.55, 9.02)	*F*_(3, 192)_ = 57.5, *p* < 0.001, η_*p*_^2^ = 0.473	ASD + = ASD− > > ADHD > TD
AQ att (95% C.I.)	18.3 (17.2, 19.4)	18.9 (16.9, 21.0)	15.0 (13.3, 16.7)	11.6 (10.4, 12.9)	*F*_(3, 192)_ = 24.5, *p* < 0.001, η_*p*_^2^ = 0.277	ASD + = ASD− > ADHD > TD
AQ det (95% C.I.)	16.4 (15.1, 17.6)	17.1 (14.9, 19.4)	15.3 (13.4, 17.2)	15.9 (14.5, 17.3)	*F*_(3, 192)_ = 0.573, *p* = 0.634, η_*p*_^2^ = 0.009	ASD + = ASD− = TD = ADHD
AQ com (95% C.I.)	19.1 (17.8, 20.3)	21.4 (19.1, 23.8)	14.6 (12.7, 16.6)	9.14 (7.72, 10.55)	*F*_(3, 192)_ = 44.5, *p* < 0.001, η_*p*_^2^ = 0.410	ASD + = ASD− > > ADHD > > TD
AQ imm (95% C.I.)	16.8 (15.6, 17.9)	17.5 (15.6, 19.5)	12.7 (11.0, 14.3)	9.81 (8.60, 11.02)	*F*_(3, 192)_ = 29.4, *p* < 0.001, η_*p*_^2^ = 0.315	ASD− = ASD+ > > ADHD > TD
EQ tot (95% C.I.)*	20.6 (18.7, 22.4)	20.0 (17.3, 22.5)	25.7 (23.6, 27.9)	35.5 (33.3, 40.0)	*F*_(149)_ = 32.1, *p* < 0.001, η_*p*_^2^ = 0.393	ASD− = ASD+ < ADHD < < TD

Covariates and estimates appearing in the model are evaluated at the following values: Age = 9.17, IQ = 98.9. Bootstrap results are based on 1,000 bootstrap samples stratified by group and computed using Bias-correction. *EQ participants: ASD+ = 24, ASD− = 70, ADHD = 32, TD = 29.

Equality of variance conditions were not met for C-AQ. Therefore, we decided to use a stratified BCa for both outcome measures to have more robust error ranges. We detected a main effect of group for both the C-AQ [*F*_(3, 192)_ = 60.0, *p* < 0.001 η*_*p*_*^2^ = 0.448] and C-EQ [*F*_(3, 149)_ = 32.1, *p* < *0.001*, η*_*p*_*^2^ = 0.393]. We found neither the age effect [*F*_(1, 192)_ = 0.60, *p* = 0.438] nor the IQ effect [*F*_(1, 192)_ = 1.21, *p* = 0.272] on C-AQ and C-EQ [*F*_(1, 149)_ = 0.253, *p* = 0.616 and *F*_(1, 149)_ = 3.10, *p* = 0.081, respectively].

**FIGURE 1:**
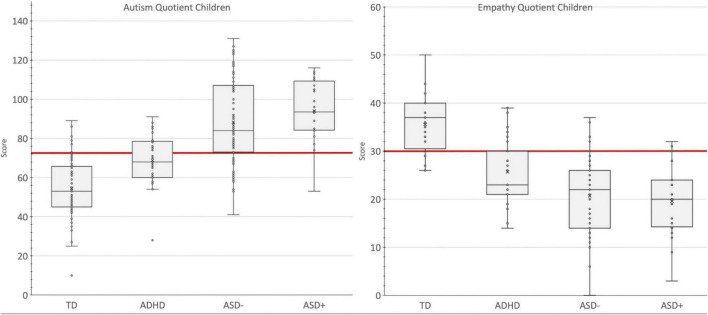
Group comparison on the C-AQ and C-EQ total scores. Box and Whisker Plot. A box is drawn from the first quartile to the third quartile, while a line is drawn at the median and the cross is the mean value. The whiskers extend from each quartile to the minimum or maximum. The Red Line is the Cut-off Value with the Highest Accuracy in Discriminating Autism Spectrum Disorder. TD, Typical Development Group; ADHD, Attention Deficit and Hyperactivity Group; ASD–, Autism Spectrum Disorder Group; ASD+, ASD with comorbid ADHD group.

On both measures, pairwise comparisons showed that the ASD+ and ASD− groups had comparable scores, with, a mean difference of 5.18 [−2.93, 14.27], *p* = 0.237, for the C-AQ and −0.52 [−5.25, 4.22], *p* = 0.787 for the C-EQ (Figure 1).

Furthermore, ASD− scores were significantly different from both the ADHD (−19.54 [−25.95, 13.25], *p* = 0.001 for the C-AQ; 5.16 [8.20, 2.07], *p* = 0.001 for the C-EQ) and the TD group (−34.17 [−40.01, −27.96], *p* = 0.001 for the C-AQ and 14.94 [11.94, 18.13], *p* = 0.001 for the C-EQ), respectively. Children with ADHD, in turn, displayed, on both the C-AQ and C-EQ, intermediate mean scores, but significantly different than TD children (14.63 [8.36, 20.61], *p* = 0.001 for the C-AQ and −9.78 [−14.84, −4.71], *p* = 0.001 for the C-EQ).

### Group Differences in Child Versions of the Autism Quotient Subscales Scores

Equality of variance conditions were not met for the social and the attention to details subscales. As before, we performed a stratified BCa. The main effect of the group, reported on the C-AQ total score, was confirmed by the multivariate analysis [*F*_(15, 570)_ = 8.49, *p* < 0.001, η_*p*_^2^ = 0.183]. We found no effect of age [*F*_(5, 188)_ = 0.719, *p* = 0.610], while IQ had a significant effect [*F*_(5, 188)_ = 4.20, *p* = 0.001, η_*p*_^2^ = 0.100]. Univariate analysis led to a main effect of group for communication [*F*_(3, 192)_ = 44.5, *p* < 0.001, η_*p*_^2^ = 0.410], social [*F*_(3, 192)_ = 57.5, *p* < 0.001, η_*p*_^2^ = 0.437], attention switching [*F*_(3, 192)_ = 24.5 *p* < 0.001, η_*p*_^2^ = 0.277], and imagination [*F*_(3, 192)_ = 29.4 *p* < 0.001, η_*p*_^2^ = 0.315] subscales. There was no group effect for the attention to details subscale [*F*_(3, 192)_ = 0.57 *p* = 0.634, η_*p*_^2^ = 0.009)]. The effect of IQ was significant for the social skills and attention to details subscales [*F*_(1, 192)_ = 5.81 *p* = 0.017, η_*p*_^2^ = 0.029 and *F*_(1, 192)_ = 4.02 *p* = 0.046, η_*p*_^2^ = 0.021, respectively].

**FIGURE 2:**
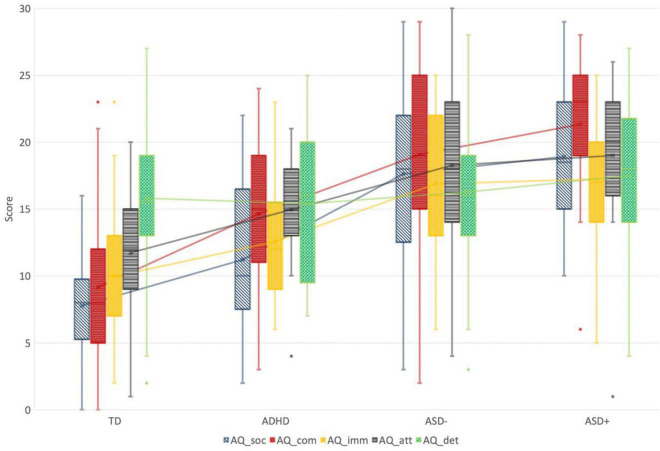
Group differences on the C-AQ subscales scores. TD, Typical Development Group; ADHD, Attention Deficit and Hyperactivity Group; ASD–, Autism Spectrum Disorder Group; ASD+, ASD with comorbid ADHD group; AAQ_soc, Autism Quotient Children—Social Skills Subscale; AQ_com, Autism Quotient Children—Communication Subscale; AQ_soc, Autism Quotient Children—Imagination Subscale; AQ_imm, Autism Quotient Children—Attention Shifting Subscale; AQ_soc, Autism Quotient Children—Attention to Details Subscale.

Pairwise comparisons showed that, on the social, communication, imagination and attention switching subscales, ASD+ and ASD− groups had comparable scores (all *p*-values > 0.386), both groups scored higher than the ADHD only and TD group (all *p*-values < 0.021), and in turn, ADHD scores were higher than the control group (all *p*-values < 0.045). There were no significant differences among the four groups on the attention to detail score (all *p-*values > 0.787). Figure 2 displays the group differences on the C-AQ subscales scores.

### Accuracy of the Child Versions of the Autism Quotient and Empathy Quotient in Predicting Group Differences

**FIGURE 3:**
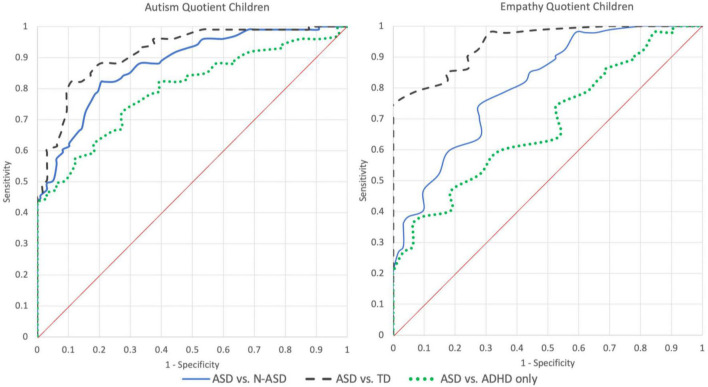
ROC curves of the C-AQ and C-EQ for different combinations of groups. TD, Typical Development Group; ADHD, Attention Deficit and Hyperactivity Group; ASD, Autism Spectrum Disorder with or without comorbid ADHD Group; N-ASD, TD and ADHD grouped together.

Using ROC curve analysis for a positive ASD diagnosis (ASD vs. N-ASD), independent of the ADHD status, the AUC was 0.877 [0.830, 0.923] for C-AQ, and 0.806 [0.737, 0.874] for C-EQ (Figure 3).

We also determined the best threshold able to discriminate between ASD and N-ASD with maximum accuracy. The cut-off was 73 (equal or higher) for the C-AQ and 26 (equal or less) for the C-EQ. Using those values, for C-AQ the sensitivity was 82% and the specificity was 79% leading to an overall accuracy of 81%, while for C-EQ the sensitivity was 75% and the specificity was 72%, with an accuracy of 73% (Table 2).

**TABLE 2:** ROC analysis and accuracy for different combination of groups.

Comparison	Scale	# Pos.	# Neg.	AUC	S.E.	Sig.	95% C.I.	Cut-off	Sensibility	Specificity	Accuracy
ASD vs. N-ASD	AQ	101	97	0.877	0.024	<0.001	0.830–0.923	73	0.822	0.794	0.808
	EQ	94	61	0.806	0.035	<0.001	0.737–0.874	26	0.745	0.721	0.733
ASD vs. ADHD	AQ	101	33	0.795	0.039	<0.001	0.719–0.872	77	0.723	0.727	0.725
	EQ	94	32	0.680	0.051	0.002	0.581–0.779	18	0.362	0.937	0.650
ASD vs. TD	AQ	101	64	0.918	0.021	<0.001	0.877–0.959	73	0.822	0.891	0.857
	EQ	94	29	0.945	0.019	<0.001	0.907–0.983	26	0.745	1.000	0.873

A comparison between ASD and TD, led to an AUC of 0.918 [0.877, 0.959] with a cut-off of 73 for AQ-C scores, corresponding to a sensitivity of 82% and a specificity of 89% with an accuracy of 86%. On the C-EQ, AUC was 0.945 [0.907, 0.983] with a cut-off of 26, a sensitivity of 75%, a specificity of 100% and an accuracy of 87%.

Finally, the direct comparison of the whole ASD group (ASD+ and ASD−) vs. the ADHD group, led, for the C-AQ, to an AUC of 0.795 [0.719, 0.872] with a cut-off of 77 corresponding to a sensitivity of 72% and a specificity of 73% with an accuracy of 73%. For the C-EQ, AUC was 0.680 [0.581, 0.779] using a cut-off of 18, sensitivity was 36% and specificity was 94% leading to an accuracy of 65%.

## Discussion

Recent literature is posing specific attention to the clinical implications of having a comorbid ASD and ADHD with a focus on investigating the extension to which the two co-occurring conditions contribute to a different phenotype expression compared to ASD and ADHD alone. Although clinical data support the presence of a more severe outcome and impairment in the quality of life, evidence from the current standardized clinical measures is still inconsistent in detecting, with sufficient accuracy, the neuropsychological differences between individuals with ASD, individuals with a comorbid ADHD (ASD+) and individuals with an ADHD alone. One of the reasons for this clinical challenge, is that, for example, many individuals with ADHD, as well as individuals with ASD, show significant impairments in the social interaction and communication area, as displayed by higher scores on the social affect domain at the Ados Diagnostic Observation Schedule, second edition (ADOS-2), and the main domain that allows to distinguishing between ASD and ADHD individuals at the ADOS-2 is based on the repetitive and restricted behaviors that remain a specific core domain for ASD (Grzadzinski et al., 2016). Similar findings have been reported when individuals with ASD and ASD+ have been compared in their social affect profile at the ADOS-2 (Harkins et al., 2021). However, other measures, based on parent-reports, such as the Social Responsiveness Scale, 2nd Edition (SRS-2) (Constantino and Gruber, 2012), demonstrated a significant group difference between children with ASD and children with ASD+, the latter group scoring significantly higher. Overall, these findings underline that there is still inconsistency in the psychological measures sensitivity and specificity to disentangle the behavioral phenotype of the two conditions both separately and even more, when associated.

To address this gap, in our study, we explored whether two quantitative measures of autism traits and empathy such as the C-AQ and C-EQ were able to distinguish children with ASD with or without ADHD, from children with ADHD alone, and children with typical development. For this reason, we used a ROC analysis to determine the threshold score that maximized classification accuracy among the conditions for each measure (Figure 3). We found that the C-AQ has good accuracy at distinguishing between ASD and N-ASD children (80%), and between ASD and TD (86%) and a satisfactory accuracy at discriminating between ASD and ADHD (73%). Our results confirm that the C-AQ is a reliable quantitative measure not only to discriminate between an ASD vs. a non-ASD condition as well as a typical development, but is also able to put apart, with a satisfactory discriminative power, an ASD from an ADHD condition. Furthermore, a cut-off of 73 was able to reliably distinguish between ASD from non-ASD conditions, while a cut-point value of 77 was the best score for discriminating ASD from ADHD. These threshold values are very similar to those reported by Auyeung et al. (2008) (cut-off of 76 for ASD vs. TD groups) confirming the cross-cultural stability of the instrument.

Conversely, the C-EQ demonstrated good accuracy at distinguishing between ASD and TD (87%) but not a sufficient accuracy in the distinction between ASD and ADHD (65%). In line with previous evidence, one possible explanation for the latter finding is that also children with ADHD may show a specific deficit in empathy (especially the affective component) as it has been reported by several studies (Abdel-Hamid et al., 2019; Harmsen, 2019; Cristofani et al., 2020; Fantozzi et al., 2021; Lasmono et al., 2021). Furthermore, this result is not unexpected, being that empathy is a trans-categorical psychological trait, which implies many aspects of social cognition, prosocial behavior, emotion regulation and morality, and is involved in different neurodevelopmental and psychiatric conditions (Henry et al., 2016; Lamm et al., 2016; Cotter et al., 2018). To account for this potential bias, from a clinical perspective, it might be considered that the cut-off of 18 (see Supplementary Table 1), maximizes specificity (94%) to the detriment of sensitivity (36%). Another possible explanation for a reduced accuracy of the C-EQ in discriminating between ASD and ADHD is that within the autism heterogeneity, a subgroup of children and individuals with ASD, for their specific clinical profile or different mechanisms of compensation and masking, do not actually score significantly lower at the empathy tasks, compared with neurotypical individuals (Rueda et al., 2015; Alkire et al., 2021; Rieffe et al., 2021). Indeed, to reach a sensibility for ASD of 90%, the cut-off on C-EQ should be raised to 32, leading to a specificity of 76% compared to TD and 22% compared to ADHD. As for the C-AQ, we also confirmed for the C-EQ good cross-cultural stability (Baron-Cohen and Wheelwright, 2004).

Univariate analysis (Figures 1, 2) revealed significant group differences between ASD, scoring the highest, ADHD, presenting intermediate scores and TD scoring the lower. This finding supports the robustness of the two measures to detect ASD traits not only in relation to typical development but also vs. other clinical conditions sharing common traits, such as ADHD. Interestingly, in our sample, children with ASD+ did not score significantly higher than children with ASD alone. A recent study by Pehlivanidis et al. (2021) reported similar findings at the AQ, showing that adults with ASD+ and ASD− had comparable scores, in turn significantly higher than the ADHD group. It means that the effect of comorbid ADHD seemed not to be additive in the reported severity of ASD and the reasons should be examined in future studies. We hypothesized two possible reasons, as follows: it might be that within the autism spectrum the psychological measures detected by the two quantitative instruments are fairly independent of ADHD traits; alternatively it could be related to a behavioral overshadowing of ASD on ADHD (for instance, if a child has difficulties in making friends due to impulsivity, but has also due to major social-communication difficulties, the latter can overshadow the first one).

### Limitations

The study has some limitations. The first limitation concerns the demographic characteristics of the sample. Furthermore, average IQ in ASD+ children was higher than in the other groups. Previous studies demonstrated that C-AQ and C-EQ are independent of age and IQ (Baron-Cohen et al., 2001, 2006; Chapman et al., 2006; Auyeung et al., 2008), Also a *post hoc* stratified boot-strapped analysis led to similar results. Secondly, we have not been able to collect ADHD-related specific measures, therefore, for the purpose of the study, we focused on the discriminative ability of quantitative, parent-report, quick screening measures such as the C-AQ and the C-EQ to detect, relatively early, an ASD condition vs. other clinical overlapping conditions such as ADHD. Furthermore, in future larger studies, cluster analysis and single item resolution analysis would be worthy to explore the specificity and transdiagnostic domains of autism traits distribution and empathy in a hybrid dimensional approach.

## Conclusion

In our study we found that the C-AQ and C-EQ are reliable and robust instruments to quantify ASD traits and empathy in children with normal intelligence and fluent language with and without comorbid ADHD as compared to children with an ADHD condition alone and TD children. Specifically, within the autism spectrum, the presence of a comorbid ADHD does not influence the severity and distribution of autistic traits and empathy, while children with a diagnosis of ADHD displayed an intermediate phenotype, with higher levels of autistic traits and lower empathy compared to TD children. The results of our study will also contribute to the translational efforts in developing better tailored treatments and preventive strategies.

## Data Availability Statement

The raw data supporting the conclusions of this article will be made available by the authors, without undue reservation.

## Ethics Statement

The studies involving human participants were reviewed and approved by the Ethics Committees of CNR (ethical clearance, 01.08.2018). Written informed consent to participate in this study was provided by the participants’ legal guardian/next of kin.

## Author Contributions

SA contributed to the study design and to the manuscript draft. DV conducted the statistical analysis and statistical data interpretations and participated in the manuscript draft. ACe contributed with statistical data interpretations and to the manuscript draft. EL, CC, FF, ACa, and FlM enrolled and tested the ASD+, ASD−, and TD participants. RS, MA, and EG enrolled and tested the ADHD participants. FrM and GT contributed with statistical analysis and statistical data interpretations. GP contributed to the coordination of the study. AG participated in the study design, coordinated the enrollment of ADHD participants and contributed to the manuscript draft. LR designed and supervised the study and drafted the manuscript. All authors read and approved the final manuscript.

## Conflict of Interest

The authors declare that the research was conducted in the absence of any commercial or financial relationships that could be construed as a potential conflict of interest.

## Publisher’s Note

All claims expressed in this article are solely those of the authors and do not necessarily represent those of their affiliated organizations, or those of the publisher, the editors and the reviewers. Any product that may be evaluated in this article, or claim that may be made by its manufacturer, is not guaranteed or endorsed by the publisher.
